# The progression of chronic tinnitus over the years

**DOI:** 10.1038/s41598-021-83068-5

**Published:** 2021-02-18

**Authors:** Jorge P. Simões, Patrick K. A. Neff, Berthold Langguth, Winfried Schlee, Martin Schecklmann

**Affiliations:** 1 Department of Psychiatry and Psychotherapy, University of Regensburg, Regensburg, Germany; 2 University Research Priority Program ’Dynamics of Healthy Aging’, University of Zurich, Zurich, Switzerland

**Keywords:** Psychology, Medical research

## Abstract

Little is known about the trajectory of tinnitus over time. This study addressed (1) how often tinnitus remitted in patients with chronic tinnitus; (2) how subjective reported tinnitus characteristics, such as loudness, laterality, and type and measures of burden, such as tinnitus distress, depression, and quality of life, changes over time; (3) how often tinnitus-specific treatments were undertaken and the prevalence of comorbidities, (4) if the number of treatments and comorbidities were associated to changes in tinnitus distress over time. Data from 388 patients with chronic tinnitus who visited a tertiary tinnitus clinic between 2012 and 2017 were interrogated via a mail survey in 2018. Tinnitus characteristics were measured with the Tinnitus Sample Case History Questionnaire and numeric rating scales; tinnitus distress with Tinnitus Handicap Inventory (THI) and the Tinnitus Questionnaire (TQ), depression with the Major Depression Inventory and Quality of life with the World Health Organisation Quality of Life BREF at both time points and the clinical global impression scale. Comorbidities experienced and undertaken treatments were assessed with an in-house survey. Three participants (0.8% of the sample) reported tinnitus remission between both assessments. A decrease in the THI and TQ, and numeric ratings for tinnitus severity, annoyance, unpleasantness, and discomfort was observed, but no differences in tinnitus characteristics, depression, quality of life or overall health status. 64% presented at least one comorbidity, and 88% sought at least on tinnitus-specific treatment. Common comorbidities were psychological and sleeping problems, and the most common interventions were going to the dentist, taking medications, and wearing hearing aids. Our results suggest that full remission of tinnitus is a rare condition, that tinnitus distress on average decreases over time, and that tinnitus characteristics, quality of life, and depression tend to remain unaltered. The high number of interventions and comorbidities displayed minimal association to the changes in tinnitus distress, highlighting the substantial and durable burden of tinnitus sufferers.

## Introduction

Tinnitus can be described as the perception of sounds, usually inthe form of ringing or hissing, without an external source^1,2^. Although a considerable number of patients report little to no impact in their daily lives due to this condition, tinnitus can also be exasperating^2,3^. Previous studies from 2005 and 2013 estimated that, at the time, 13 million tinnitus patients were actively seeking support in Europe and in the United States^4,5^. Furthermore, the costs associated with tinnitus were estimated at 750 million pounds per year in the United Kingdom^6^. Overall, tinnitus can be described as a burdensome condition both at the individual and societal level.

The reasons why only a subset of patients are burdened by the condition are unclear. Moreover, evidence suggests tinnitus can change over time even when presented chronically^7^. Based on clinical experience it is generally assumed that tinnitus becomes less burdensome over time, but systematic data supporting this assumption are scarce. One mechanism of amelioration of tinnitus over time may be related to tinnitus habituation, first described by Hallam^8^. It is also a key component of the so-called neurophysiological tinnitus model, and it offers a potential explanation why the condition is so burdensome for a subset of patients^9^. According to this model, distress originates from aberrant activation in areas of the brain other than the auditory pathway, such as the limbic and autonomic systems, which prevents that patients habituate to tinnitus by repeated emotional and stressful ratings of the condition^10,9^. This concept is supported by recent imaging data demonstrating the involvement of non-auditory brain areas such as the frontal cortex, the amygdala and the parahippocampal cortex in tinnitus pathophysiology^11^.

Recent studies using ecological momentary assessment indicate that tinnitus distress fluctuates over time and that these fluctuations are related to emotional factors^12,13^. However, very little is known about the course of tinnitus over several years and about the facets of tinnitus that might vary over time, even if this knowledge would be of exceptional interest for clinicians, researchers and patients^14,15^.

In the following, some of the longitudinal studies, alongside their main findings and limitations are briefly discussed. Gr iest and Bishop^16^ conducted a 15-year longitudinal study on noise-exposed workers in which the authors identified tinnitus as an early indicator of hearing loss. In a 7-year longitudinal study, Andersson et al.^17^ found that tinnitus severity shows signs of improvement over time after clustering patients in three groups based on different degrees of distress. However, the authors did not use any distress-specific, numeric questionnaire to compare baseline and follow-up values. Olderog and colleagues^18^ conducted a study with 44 patients suffering from tinnitus for less than four weeks with a 6-month follow-up survey. A stepwise regression with the predictors “sleep disturbance”, “anxiousness”, and “life satisfaction” collected at baseline could explain 56% of the variance (R2) of tinnitus distress measured with the Tinnitus Questionnaire (TQ) in the 6-month follow up. It remains unclear, however, whether these effects would persist in a larger, chronic tinnitus sample. Erlandsson and Persson^19^ found that the Beck Depression’s Inventory (BDI) and the trait and state anxiety (quantified by Spielberger’s State and Trait Anxiety Inventory) decreased over a period of 18 months only among patients without a personality disorder. Lastly, Folmer^20^ measured sleep quality, depression (measured by the BDI), and tinnitus distress (measured by the Tinnitus Severity Index, TSI) in 190 patients. The author reported a decrease in the TSI, especially among patients whose sleep patterns improved and whose BDI decreased by at least 3 points between the two assessments.

We investigated whether tinnitus changes over time in a large cohort of patients with chronic tinnitus from a tertiary clinic in a retrospective mail survey to further understand the development of tinnitus over time. Our research questions were (1) how often tinnitus remits in patients with chronic tinnitus; (2) how tinnitus characteristics and burden caused by tinnitus (tinnitus distress, depression, quality of life and health status) change over time; (3) how often patients enrolled in a tinnitus-related treatment protocol and which treatments were most often sought; (4) if number of treatments or comorbidities were associated with changes in tinnitus distress over time.

## Results

Three patients (0.8%) reported losing their tinnitus at T2. Table 1 summarises the demographics of our sample compared to those three individuals. Overall, we did not observe any clear pattern that could explain why those patients lost their tinnitus.

**Figure 1:**
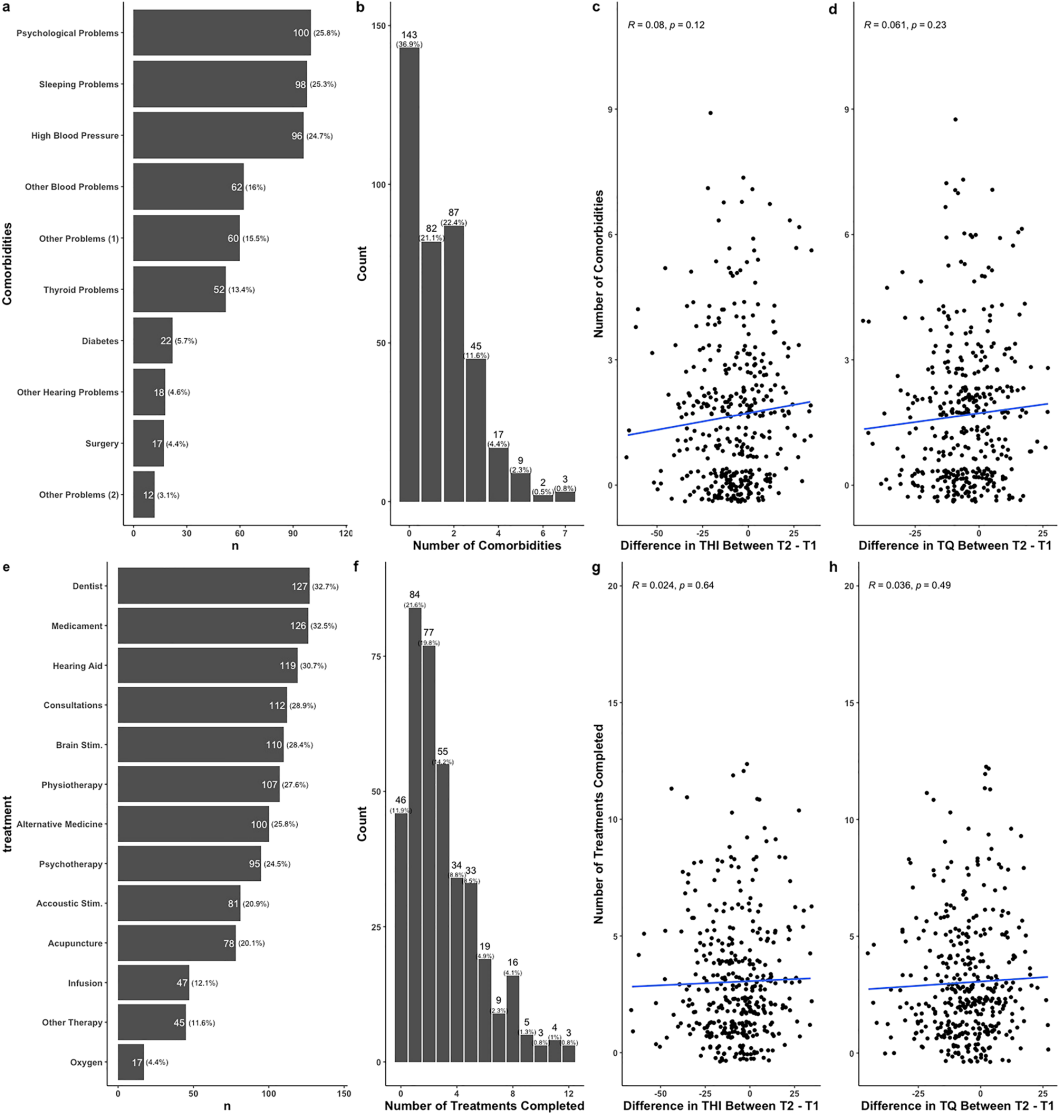
Relation between the number of comorbidities experienced, treatments tried, THI and TQ over time.

Table 2 presents the difference between T1 and T2. We observed significant differences between the two time points for the THI, TQ and for numeric ratings for tinnitus severity, its discomfort, its annoyance, and its unpleasantness. For all other variables we did not find a significant difference between T1 and T2. Regarding the THI, 87 participants (22% of the sample) reported a decrease greater than 20 points between the two time points, while 182 (47%) reported a decrease in the THI of at least 7 points. Appointment to the dentist was the most frequent treatment patients sought for their tinnitus (32.7%), followed by medication (32.4%), and hearing aids (30.6%, Fig. 1e), and most patients (88%) tried at least one treatment for their tinnitus (Fig. 1f).

**Figure 2:**
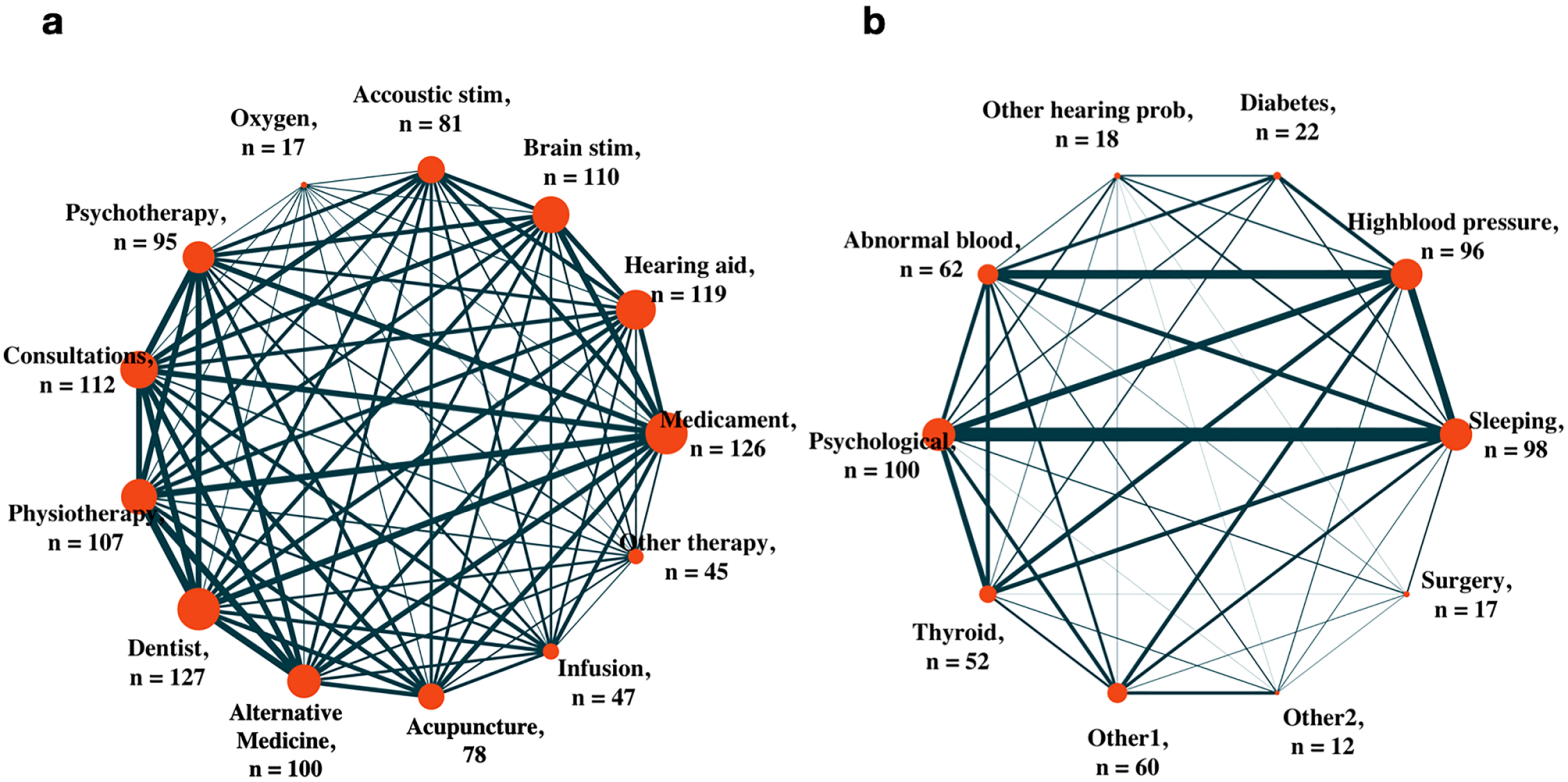
Network depiction of the relation between treatments (**a**) and comorbidities (**b**). The prevalence of comorbidities and the number of treatments undertaken by patients are represented by the size of the node. The link represents a pair of comorbidities experienced or treatments tried by a given patient, and its thickness represents the number of patients who experienced both comorbidities or sought both treatments.

Most patients (64%) reported having at least one comorbidity, with psychological and sleeping problems being the two most often reported (Fig. 1a,b). Visiting the dentist and taking medication was the two most common treatments tried by patients between T1 and T2 (Fig. 1e), and most patients (88%) tried at least one treatment for their tinnitus (Fig. 1f). Figure 2 shows a network representation of the relation between treatments (Fig. 2a) and comorbidities (Fig. 2b) experienced by patients between T1 and T2. Interestingly, the two most common comorbidities, psychological and sleeping problems, showed the strongest association (Fig. 2b, as depicted by the link’s thickness). Next, we investigated whether the number of comorbidities experienced, and the number of treatments tried between T1 and T2 influences tinnitus distress changes over time, measured as $\Delta$THI and $\Delta$TQ (Fig. 1). Figure 1g,h show a correlation between the number of treatments tried and $\Delta$THI (r = 0.08, *p* = 0.12) and $\Delta$TQ (r = 0.06, *p* = 0.23) respectively, whereas Fig. 1c,d show no significant correlation between the number of comorbidities experienced by patients between T1 and T2 and $\Delta$THI (r = 0.024, *p* = 0.64) and $\Delta$TQ (r = 0.04, *p* = 0.49) respectively.

## Discussion

We analysed retrospective longitudinal data of patients with chronic tinnitus from a tertiary tinnitus clinic. 388 of the 1213 contacted patients responded to the mail, resulting in a response rate comparable to a previous study with a cohort from the same clinic^21^. Three patients (0.8% of our sample) reported full remission of tinnitus at T2. No common factors or characteristics were identified distinguishing those patients from the whole sample (Table 1). It is often suggested that tinnitus remits especially in its acute presentation^3,22^. Our results suggest that, albeit rare, tinnitus may also disappear in chronic patients suffering from the condition for years or even decades. These numbers, however, may be underestimated as other patients who also lost their tinnitus may have not responded to the survey. We were not able to further investigate potential factors associated with remission as patients did not agree to be further contacted. Future studies could focus on this select subgroup of patients with prospective follow-up studies from large cohorts. If investigators choose to focus on remission per se, then patients with acute tinnitus, in which the remission rate is higher, could be studied^22^.

Tinnitus distress when measured using the THI and TQ, and the tinnitus severity items regarding the condition as troublesome, uncomfortable, annoying and unpleasant decreased over time (Table 2). Regarding the THI, 22.4% of patients reported a decrease of 20 or more points between the two time points, and 46.9% reported a decrease of 7 or more points. Those two cut-off points have been previously suggested to represent “clinical meaningful improvements” of tinnitus^23,24^, but empirical evidence supporting their usage is scarce. Other measures of burden, such as depression, quality of life or overall health status did not change between the time points. If changes in tinnitus distress do not impact the daily life of the patients, it is to discuss in what way the reported changes are meaningful. There is increasing discussion in the field of tinnitus and other conditions that quality of life should have more awareness in tinnitus trials^25^. Future studies could evaluate the clinical relevance of these two definitions of “clinical improvement” from the THI based on whether they can distinguish improvements in quality of life and/or depression, for example.

Additionally, there was no difference in tinnitus characteristics, such as loudness, type of perceived sound, and laterality between the two time points after controlling for multiple comparisons. These results suggests that the acoustic perception remains largely stable over time, but the distress diminishes, e.g. by habituation. In this context, an important aspect of future research is the identification of factors that facilitate distress reduction. A previous study using the same cohort as the one from this study indicated that the personality traits neuroticism and extraversion are respectively negatively and positively related to $\Delta$THI and $\Delta$TQ^26^.

The high number of treatments patients sought, and the high number of comorbidities experienced highlight the burden tinnitus may cause^6^. However, we observed no linear relation between number of treatments and tinnitus distress. It has been proposed that tinnitus treatment should be based on precision medicine, as not all treatments are equally beneficial to patients^27,28^. Both clinicians and patients have indicated the lack of an universal treatment for tinnitus as one of their biggest complaints^29^. However, these results may be confounded by a selection bias, as patients not severely affected by tinnitus may have improved after a first treatment or no treatment whatsoever. Since patients were not randomised into treatment or no treatment groups, our study can not quantify the potential effects of treatments on the longitudinal trajectory of tinnitus distress. The same considerations apply when interpreting the relation between comorbidities and tinnitus distress.

Our analysis was limited to factors such as demographics, tinnitus distress and tinnitus characteristics, quality of life and depression. However, other confounding variables such as socio-demographics, coping strategies, life history, and personality^26^ could be of central interest for the longitudinal trajectory of tinnitus. Another limitation of our study was the variability of the time point when patients were first assessed (i.e., between 2012 and 2017) and the variability of treatments and comorbidities included in this analysis. Only a subset of patients reported to our mail survey; therefore, the possibility of a selection bias must be considered. Additionally, the observed improvement might reflect the tendency to the mean, that is, patients visit the clinic when they are severely impaired, and a later improvement may reflect spontaneous fluctuations. Future studies should assess both subjective and objective (e.g., minimum masking level, tinnitus matching, etc.) tinnitus characteristics at all time points.

**Table 1:** Characteristics of our sample at T1.

	Mean (SD), N = 385	Patient #1	Patient #2	Patient #3
Gender$^{1}$	146/242	Female	Female	Male
Age (years) [SD]	55.9 [11.9]	51	64	63
Duration (months) [SD]	154.3 [104.1]	77	266	158
Type of Tinnitus sound*	232/31/72/46	Tone	Crickets	Tone
Laterality of Tinnitus**	50/62/88/57/85/41	Right ear	Both ears	Left ear
Loudness (1–100) [SD]	68.2 [53.3]	20	90	70
MDI (mean total score) [SD]	15.3 [11.5]	NA	22	14
THI (mean total score) [SD]	49.5 [23.3]	54	72	52
TQ (mean total score) [SD]	42.2 [17.7]	33	64	49

The characteristics of all three patients who reported losing their tinnitus between the two assessments is presented. 1: female/male ratio.

* from left to right: Tonal, Noise, Crickets, Other. ** from left to right: right ear, left ear, both ears (worse in right), both ears (worse in left), both ears (equally bad), inside the head. NA: Non Available.

**Table 2:** Differences between T1 and T2.

Domain	Subscore	Mean [sd] / Ratio at baseline	Mean [sd]/Ratio at 2nd assessment	Test [ci]	*p* value	Adjusted p	Effect Size
Distress	Problem*	2.55 [0.9]	2.27 [1.1]	4.33 [0.2–0.4]	0.001	0.001	0.33
	Loudness*	6.73 [2.1]	6.37 [2.5]	2.3 [0.1–0.7]	0.02	0.13	0.17
	Loudness***	68.73 [58.28]	63.61 [22.05]	1.66	0.09	0.49	0.12
	Uncomfortableness*	7.4 [2.3]	6.72 [2.6]	4.02 [0.4–1.01]	0.001	0.001	0.3
	Annoyance*	7.11 [2.4]	6.39 [2.7]	3.99 [0.4–1.1]	0.001	0.001	0.29
	Ignore*	7.18 [2.6]	6.72 [2.9]	2.37 [0.1–0.8]	0.02	0.13	0.18
	Unpleasantness*	7.17 [2.3]	6.47 [2.7]	4.01 [0.4–1.1]	0.001	0.001	0.3
	THI	49.54 [23.1]	41.98 [23.8]	4.38 [4.2–10.9]	0.001	0.001	0.32
	TQ	42.31 [17.7]	36.87 [19.1]	4.06 [2.8–8.1]	0.001	0.001	0.3
Depression	MDI	15.28 [11.5]	13.51 [10.4]	2.07 [0.1–3.5]	0.04	0.19	0.16
Quality of life	Physical health**	12.72 [1.7]	12.89 [1.7]	$-$ 1.39 [$-$ 0.4–0.07]	0.16	0.59	$-$ 0.1
	Psychological**	13.72 [2.1]	13.73 [2.1]	$-$ 0.09 [$-$ 0.3–0.3]	0.93	1	$-$ 0.01
	Social relationships**	14.6 [3.3]	14.25 [3.3]	1.45 [$-$ 0.1–0.8]	0.15	0.59	0.11
	Environment**	16.7 [2.2]	16.6 [2.2]	0.62 [$-$ 0.2–0.4]	0.54	1	0
Tinnitus characteristics***	Begin of perception$^{1}$	175/185	175/185	0	1	1	0
	Pulsation$^{2}$	43/36/287	35/42/289	1.29	0.53	1	0.06
	Laterality of Tinnitus$^{3}$	48/59/80/55/78/38	34/59/80/53/82/50	4.16	0.52	1	0.11
	Intermittence$^{4}$	41/336	63/314	5.4	0.03	0.66	0.12
	Loudness fluctuation$^{5}$	236/136	237/135	0.01	1	1	0
	Tone/white noise$^{6}$	220/27/64/44	214/44/65/32	6.06	0.11	1	0.13
	Tone frequency$^{7}$	114/191/59/4	116/175/72/5	2.12	0.56	1	0.08
	React to positive sounds$^{8}$	238/84/49	253/70/48	1.74	0.43	1	0.07
	React to negative sounds$^{9}$	191/84	196/79	0.22	0.71	1	0.03
	Somatic component$^{10}$	146/226	141/231	0.14	0.77	1	0.22
	Effect of nap$^{11}$	60/30/258	56/40/252	1.64	0.44	1	0.07
	Effect of sleep$^{12}$	77/121/157	89/125/141	1.79	0.4	1	0.07
	Effect of strees$^{13}$	261/4/100	275/6/84	2.16	0.34	1	0.08
	Hearing problem$^{14}$	232/137	241/128	0.48	0.54	1	0.04
	Wear hearing aids$^{15}$	8/9/48/304	11/13/70/275	6.76	0.08	1	0.14
	Toleration to loudness$^{16}$	38/47/152/69/69	21/61/131/85/77	10.37	0.03	0.73	0.17
	Hyperacusis$^{17}$	185/117	203/99	2.34	0.15	1	0.09
	Headache problems$^{18}$	158/212	119/251	8.78	0	0.08	0.15
	Dizziness/vertigo$^{19}$	132/231	104/259	4.92	0.03	0.73	0.12
	TMJ$^{20}$	93/275	91/277	0.03	0.94	1	0.01
	neck pain$^{21}$	235/138	209/164	3.76	0.06	1	0.1
	Pain syndromes$^{22}$	170/206	163/213	0.26	0.65	1	0.03
	Psychological treatment$^{23}$	77/299	63/313	1.72	0.23	1	0.07
	Year of visit (2012–2017)	70/96/70/33/57/62	–	–	–	–	–

T-tests and chi-squared tests were used for numeric and categorical items, respectively. The last row shows the frequency of visits per year. Multiple comparisons were corrected with the Holm–Bonferroni method, and effect sizes were calculated with Cohen’s d. Missing observations in pairwise comparisons were excluded from the analysis. * Items of the Tinnitus Numeric Rating Scale; ** Subscores of WHOQoL; *** Items of Tinnitus Sample Case History Questionnaire (TSCHQ). From left to right: 1: gradual/abrupt; 2: yes, with the heart rate/yes, different from heart rate/no; 3: right ear/left ear/ both ears, worse on left/both ears, worse on right/both ears equally/inside the head/elsewhere; 4: intermitent/constant; 5: fluctuates/constant; 6: tone/noise/cricket/other sound; 7: very high freq./high freq./medium freq./low freq.; 8: yes/no/do not know; 9: yes/no; 10: yes/no/do not know; 11: worsens tinnitus/improves tinnitus/no effect; 12: no correlation between sleep and tinnitus/correlation between sleep and tinnitus/do not know; 13: stress influences tinnitus/stress does not influence tinnitus; 14: has hearing problem/does not have hearing problems; 15: right ear/left ear/both ears/none; 16: never/rarely/sometimes/usually/always; 17: suffers from hyperacusis/ does not suffer from hypearcusis; 18: suffers from headaches/does not suffer from headaches; 19: suffers from dizziness/does not suffer from dizziness; 20: suffers from tempomandibular disorders/does not suffer from tempomandibular disorder ; 21: suffers from neck pain/ does not suffer from neck pain; 22: suffers from pain syndrome/does not suffer from pain syndrome; 23: currently having psychological treatment/currently not having psychological treatment. THI: tinnitus handicap inventory; TQ: tinnitus questionnaire; MDI: major depression inventory; TMJ: transcranial magnetic stimulation.

## Conclusion

We investigated the longitudinal trajectory of tinnitus characteristics, tinnitus distress, depression and quality of life among chronic patients from a tertiary tinnitus clinic. Tinnitus disappeared from three chronic patients, suggesting that, albeit rare, remission is possible even after years of having tinnitus. Future research should further investigate which factors are associated with tinnitus remission. Our findings should also encourage future research to focus not only on tinnitus management but also on interventions to suppress it, as tinnitus remission may be possible also in the chronic manifestation of the condition.

## Methods

### Participants

Our sample consisted of patients from the Tinnitus Center of the University of Regensburg. Participants were invited to this study via a letter containing questionnaires and a consent form. The letters were sent to 1213 out-patients who visited the clinic between 2012 and 2017. 388 letters were sent back, resulting in a 32% response rate. The study was approved by the ethical committee at the faculty of medicine of the University of Regensburg (study number 18-1041-101). All the protocols and guidelines of the institution were followed, and only patients from whom we obtained signed informed consent were included in the final analysis.

### Data collection

Henceforward, the patients’ first visit to the clinic between 2012 and 2017 will be described as T1, and the assessment through mail conducted in 2018 will be described as T2. The Tinnitus Handicap Inventory (THI), Tinnitus Questionnaire (TQ), Tinnitus Numeric Ratings Scales for severity, loudness etc., Major Depression Inventory (MDI), World Health Organization Quality of Life(^30^, WHOQOL-Bref), health status according to Clinical Global Impression and the Tinnitus Sample Case History Questionnaire(^31^ TSCHQ) were collected at T1 and T2, and an informal in-house questionnaire asking patients which treatments they tried, and which comorbidities they experienced since T1 was obtained at T2 (available in the supplementary material).

### Statistical analysis

Differences between T1 and T2 (T2–T1) were calculated and reported with a $\Delta$ symbol. All statistical analyses were conducted with R statistical software (version 3.4.4,^32^), alongside the ”tidyverse” package^33^. After checking whether variables presented normal distributions and homogeneous variance, t-tests were used to compare mean differences in numeric items between T1 and T2; categorical items were tested with Pearson’s Chi-Squared test. Two previous studies defined an improvement in tinnitus distress, measured by the THI, to be clinically significant if above 20 and 7 points^23,24^. Differences in the THI between T1 and T2 were used to calculate the percentage of patients who clinically improved under these definitions. Missing observations in pairwise comparisons were excluded from the analysis. Multiple comparisons were corrected with the Holm-Bonferroni method^34^. Following convention, p values below the threshold 0.05 were considered statistically significant and p values below 0.1 were considered trend significant. Effect sizes were calculated with the Cohen’s d using the ”effsize” package^35,36^. The package Igraph was used to represent the association between comorbidities and treatments tried as a network^37^. In this context, the node’s size represents the number of people who experienced a comorbidity or attempted a treatment protocol, and the link’s thickness between two nodes represents the number of people who experienced a given pair of comorbidities or two attempted treatment protocols between T1 and T2.

## Supplementary Information

Supplementary Information 1.

Supplementary Information 2.
