# Supplementary material for: The progression of chronic tinnitus over the years
Source: Sci Rep. 2021 Feb 18;11:4162. doi: 10.1038/s41598-021-83068-5 (PMC7892997; doi:10.1038/s41598-021-83068-5)
Supplement: Supplementary file 1 — Supplementary Information 1. [file 41598_2021_83068_MOESM1_ESM.docx]

**Supplementary Material**

**The Progression of Chronic Tinnitus Over the Years**

**Authors:**

**Jorge P. Simões**

Department of Psychiatry and Psychotherapy, University of Regensburg

**Patrick K.A. Neff**

Department of Psychiatry and Psychotherapy, University of Regensburg
University Research Priority Program ’Dynamics of Healthy Aging’, University of Zurich, **Switzerland.**

**Berthold Langguth**

Department of Psychiatry and Psychotherapy, University of Regensburg

**Daria Fahramand**

Department of Psychiatry and Psychotherapy, University of Regensburg

**Winfried Schlee**

Department of Psychiatry and Psychotherapy, University of Regensburg

**Martin Schecklmann**

Department of Psychiatry and Psychotherapy, University of Regensburg

**Langzeitverlauf des chronischen Tinnitus**

1. **Bitte beantworten Sie jeden Fragebogen, auch wenn Sie ihn bei Ihrem letzten Besuch ausgefüllt haben.**
2. **Verändern Sie bitte nicht die Frage oder Antwortmöglichkeit!**
3. **Bitte kreuzen Sie die zutreffende Antwort an (⌧) oder füllen Sie leere Felder aus.**
4. **A.) Haben Sie noch Tinnitus (auch wenn Sie sich vielleicht besser ablenken oder Tinnitus besser ignorieren können)?**

JA  NEIN

1. **Bitte vergleichen Sie Ihren jetzigen Gesundheitszustand mit Ihrem Zustand bei Ihrem ersten Aufenthalt in der Sprechstunde bei uns in Regensburg am XX.XX.XXXX und schätzen Sie, um wie viel sich Ihr Tinnitus verbessert hat?**
2. Sehr viel besser
3. Viel besser
4. Etwas besser
5. Keine Veränderung
6. Etwas schlechter
7. Viel schlechter
8. Sehr viel schlechter
9. **Fühlen Sie sich über Tinnitus informiert?**

JA  NEIN

1. **Behandlungen Ihres Tinnitus**. Bitte kreuzen Sie Zutreffendes an.

| **Welche Behandlung haben Sie in der Zeit nach ihrem Erstgespräch am XX.XX.XXXX wahrgenommen?** | **Spezifizieren Sie die Art der Therapie, z.B. wenn Sie eine Medikation bekommen haben, um welches Medikament handelt es sich?** | **Dauert diese Behandlung an?** | **Bitte beurteilen Sie den Effekt der Behandlung Ihres Tinnitus.**  **1. sehr viel schlechter**  **2. schlechter**  **3. kein Effekt**  **4. besser**  **5. sehr viel besser** |
| --- | --- | --- | --- |
| medikamentöse Therapie |  | JA  NEIN | 1. 2. 3. 4. 5. |
| Hörgerät |  | JA  NEIN | 1. 2. 3. 4. 5. |
| Hirnstimulation wie z.B. TMS/Magnet-stimulation |  | JA  NEIN | 1. 2. 3. 4. 5. |
| Akustische Stimulation, Geräuschtherapie über Hörgerät, Musiktherapie, Masker |  | JA  NEIN | 1. 2. 3. 4. 5. |
| Sauerstofftherapie |  | JA  NEIN | 1. 2. 3. 4. 5. |
| Psychotherapie/ Verhaltenstherapie |  | JA  NEIN | 1. 2. 3. 4. 5. |
| Beratungsgespräche bezüglich Tinnitus |  | JA  NEIN | 1. 2. 3. 4. 5. |
| Physiotherapie |  | JA  NEIN | 1. 2. 3. 4. 5. |
| zahnärztliche Behandlung |  | JA  NEIN | 1. 2. 3. 4. 5. |
| alternative Medizin (Heilpraktiker, Osteopathie, Chiropraktiker) |  | JA  NEIN | 1. 2. 3. 4. 5. |
| Akupunktur |  | JA  NEIN | 1. 2. 3. 4. 5. |
| Infusionen |  | JA  NEIN | 1. 2. 3. 4. 5. |
| andere Therapie:  ___________________ |  | JA  NEIN | 1. 2. 3. 4. 5. |

1. **Weitere Erkrankungen.** Bitte kreuzen Sie Zutreffendes an.

| **Wurde zwischen Ihrer Erstvorstellung im Regensburger Tinnituszentrum und dem jetzigen Zeitpunkt auch eine andere Erkrankung behandelt (außer Tinnitus)?** | **Erhalten oder erhielten Sie eine medikamentöse Behandlung?** | **Bitte beurteilen Sie den Effekt der Behandlung Ihres Tinnitus.**  **1. sehr viel schlechter**  **2. schlechter**  **3. kein Effekt**  **4. besser**  **5. sehr viel besser** |
| --- | --- | --- |
| Schlafstörungen | JA  NEIN | 1. 2. 3. 4. 5. |
| erhöhter Blutdruck | JA  NEIN | 1. 2. 3. 4. 5. |
| Diabetes | JA  NEIN | 1. 2. 3. 4. 5. |
| andere Hörerkrankungen | JA  NEIN | 1. 2. 3. 4. 5. |
| erhöhte Blutwerte, z.B. Cholesterin | JA  NEIN | 1. 2. 3. 4. 5. |
| psychische Erkrankung, z.B. Depression, Angststörung | JA  NEIN | 1. 2. 3. 4. 5. |
| Schilddrüsenerkrankung | JA  NEIN | 1. 2. 3. 4. 5. |
| andere Erkrankung:  _________________________ | JA  NEIN | 1. 2. 3. 4. 5. |
| andere Erkrankung:  _________________________ | JA  NEIN | 1. 2. 3. 4. 5. |
| andere Erkrankung:  _________________________ | JA  NEIN | 1. 2. 3. 4. 5. |
| Operation:  _________________________ |  | 1. 2. 3. 4. 5. |
| Operation:  _________________________ |  | 1. 2. 3. 4. 5. |
| Operation:  _________________________ |  | 1. 2. 3. 4. 5. |

1. **Gab es in der Zeit seit Ihrer Erstvorstellung am XX.XX.XXXX Veränderungen in Ihrer Lebenssituation?**
   1. **Berufliche Veränderungen**  JA  NEIN

6.1.1 Wenn JA, war diese Veränderung  POSITIV
  NEUTRAL
  NEGATIV

**6**.**2 Private Veränderungen**  JA  NEIN

6.2.1 Wenn JA, war diese Veränderung  POSITIV
  NEUTRAL
  NEGATIV

1. **Wie ist der Tinnitus im Vergleich zu Ihrer Erstvorstellung?**

________________________________________________________________________________________________________________________________________________________________________________________________________________________________________________________________________________________________________________________________________

1. **Haben Sie einige Behandlungen aus Frage 4 zeitgleich angewendet? Wenn ja, um welche Behandlungen handelte es sich?**

______________________________________________________________________________________________________________________________________________________________________________________________________________________________________________________

1. **Gibt es Faktoren, von denen Sie glauben, sie beeinflussen den Tinnitus? Wird der Tinnitus dadurch besser oder schlechter?**

________________________________________________________________________________________________________________________________________________________________________________________________________________________________________________________________________________________________________________________________________
