# Supplementary material for: The progression of chronic tinnitus over the years
Source: Sci Rep. 2021 Feb 18;11:4162. doi: 10.1038/s41598-021-83068-5 (PMC7892997; doi:10.1038/s41598-021-83068-5)
Supplement: Supplementary file 2 — Supplementary Information 2. [file 41598_2021_83068_MOESM2_ESM.docx]

**Supplementary Material**

**The Progression of Chronic Tinnitus Over the Years**

**Authors:**

**Jorge P. Simões**

Department of Psychiatry and Psychotherapy, University of Regensburg

**Patrick K.A. Neff**

Department of Psychiatry and Psychotherapy, University of Regensburg
University Research Priority Program ’Dynamics of Healthy Aging’, University of Zurich, **Switzerland.**

**Berthold Langguth**

Department of Psychiatry and Psychotherapy, University of Regensburg

**Daria Fahramand**

Department of Psychiatry and Psychotherapy, University of Regensburg

**Winfried Schlee**

Department of Psychiatry and Psychotherapy, University of Regensburg

**Martin Schecklmann**

Department of Psychiatry and Psychotherapy, University of Regensburg

**Long term course of chronic tinnitus**

1. **Please answer each questionnaire, even if you filled it out during your last visit.**
2. **Please do not change the question or answer option!**
3. **Please tick the appropriate answer (⌧) or fill in empty fields.**
4. **A.) Do you still have tinnitus (although you may find it easier to distract yourself or ignore tinnitus)?**

YES NO

1. **Please compare your current state of health with your condition during your first visit to our office in Regensburg on XX.XX.XXXX and estimate how much your tinnitus has improved?**
2. Very much better
3. Much better
4. Somewhat better
5. No change
6. Slightly worse
7. Much worse
8. Very much worse
9. **Do you feel informed about tinnitus?**

YES NO

1. **Treatments for your tinnitus**. Please tick the appropriate box.

| **What treatment did you experience in the time after your initial consultation on XX.XX.XXXX?** | **Specify the type of therapy, e.g. if you have been given a medication, which medication is it?** | **Does this treatment last?** | **Please evaluate the effect of treating your tinnitus.**  **1. very much worse**  **2. worse**  **3. no effect**  **4. better**  **5. very much better** |
| --- | --- | --- | --- |
| drug therapy |  | YES NO | 1. 2. 3. 4. 5. |
| hearing aid |  | YES NO | 1. 2. 3. 4. 5. |
| Brain stimulation such as TMS/magnetic stimulation |  | YES NO | 1. 2. 3. 4. 5. |
| Acoustic stimulation, noise therapy with hearing aids, music therapy, mask |  | YES NO | 1. 2. 3. 4. 5. |
| Oxygen therapy |  | YES NO | 1. 2. 3. 4. 5. |
| Psychotherapy/behavioral therapy |  | YES NO | 1. 2. 3. 4. 5. |
| Counseling sessions regarding tinnitus |  | YES NO | 1. 2. 3. 4. 5. |
| physiotherapy |  | YES NO | 1. 2. 3. 4. 5. |
| Dental treatment |  | YES NO | 1. 2. 3. 4. 5. |
| alternative medicine (alternative practitioner, osteopathy, chiropractor) |  | YES NO | 1. 2. 3. 4. 5. |
| Acupuncture |  | YES NO | 1. 2. 3. 4. 5. |
| Infusions |  | YES NO | 1. 2. 3. 4. 5. |
| other therapy:  ___________________ |  | YES NO | 1. 2. 3. 4. 5. |

1. **Other diseases.** Please tick as appropriate.

| **Has any other condition (other than tinnitus) been treated between your first presentation at the Regensburg Tinnitus Center and the present time?** | **Are you receiving or have you been receiving drug treatment?** | **Please evaluate the effect of treating your tinnitus.**  **1. very much worse**  **2. worse**  **3. no effect**  **4. better**  **5. very much better** |
| --- | --- | --- |
| Insomnia | YES NO | 1. 2. 3. 4. 5. |
| high blood pressure | YES NO | 1. 2. 3. 4. 5. |
| Diabetes | YES NO | 1. 2. 3. 4. 5. |
| other hearing disorders | YES NO | 1. 2. 3. 4. 5. |
| elevated blood values, e.g. cholesterol | YES NO | 1. 2. 3. 4. 5. |
| mental illness, e.g. depression, anxiety disorder | YES NO | 1. 2. 3. 4. 5. |
| Thyroid gland disease | YES NO | 1. 2. 3. 4. 5. |
| other disease:  _________________________ | YES NO | 1. 2. 3. 4. 5. |
| other disease:  _________________________ | YES NO | 1. 2. 3. 4. 5. |
| other disease:  _________________________ | YES NO | 1. 2. 3. 4. 5. |
| Operation:  _________________________ |  | 1. 2. 3. 4. 5. |
| Operation:  _________________________ |  | 1. 2. 3. 4. 5. |
| Operation:  _________________________ |  | 1. 2. 3. 4. 5. |

1. **Have there been any changes in your life situation in the time since your first performance on XX.XX.XXXX?**
   1. **Professional changes** YES NO

6.1.1 If YES, this change was POSITIVE NEUTRAL NEGATIVE

**6**.**2 Private changes** YES NO

6.2.1 If YES, this change was POSITIVE NEUTRAL NEGATIVE

1. **How is the tinnitus compared to your initial presentation?**

________________________________________________________________________________________________________________________________________________________________________________________________________________________________________________________________________________________________________________________________________

1. **Did you apply some of the treatments from question 4 at the same time? If so, which treatments were they?**

__________________________________________________________________________________ ____________________________________________________________________________________________________________________________________________________________________

1. **Are there factors that you think influence the tinnitus? Does it make the tinnitus better or worse?**

____________________________________________________________________________________________________________________________________________________________________ ____________________________________________________________________________________________________________________________________________________________________
